# Efficacy of pectoserratus plane block versus erector spinae plane block on acute and chronic pain after mastectomy: randomized clinical trial

**DOI:** 10.1016/j.bjane.2025.844691

**Published:** 2025-10-24

**Authors:** Fabrício T. Mendonça, Marcus Alexandre B. de Aviz, Ana Paula S. Bezerra, Lucas G. Silva, Estefane E. Gaspar, Bárbara N. Terol, Lucianna R. e Silva, Liliana M. Andrade

**Affiliations:** Hospital de Base do Distrito Federal, Departamento de Anestesiologia, Brasília, DF, Brazil

**Keywords:** Analgesia, Chronic pain, Mastectomy, Nerve block, Opioid

## Abstract

**Objectives:**

To compare Pectoserratus Plane Block (PSPB) and Erector Spinae Plane (ESP) block regarding perioperative opioid consumption and chronic pain risk among women undergoing elective mastectomy.

**Methods:**

Single-blind (patients), randomized (1:1) trial. The primary outcome was the composite measure defined as the use of fentanyl intraoperatively or tramadol postoperatively. Secondary outcomes encompassed intraoperative hemodynamics, short (24h), medium (3 months) and long-term (6 months) postoperative pain intensity and complications.

**Results:**

99 patients were randomized (50 in the PSPB group and 49 in the ESP block group). Of these, 93 patients had complete data for the primary outcome. Use of either fentanyl or tramadol was required for 20 of 47 patients (43%) in the PSPB group and 28 of 46 patients (61%) in the ESP block group (Relative Risk [RR] 0.70, 95% Confidence interval [95% CI] 0.47‒1.05, p = 0.09). PSPB-treated patients had a lower risk of tramadol (RR = 0.31, 95% CI 0.12-0.77, p = 0.01) and dipyrone (RR = 0.60, 95% CI 0.39‒0.92, p = 0.02) consumption than ESP block-treated patients. PSPB lowered chronic pain risk at 3 months (RR = 0.66, 95% CI 0.47‒0.92, p = 0.02), with lower scores for the Short-Form McGill Pain Questionnaire (Mean Difference [MD] -2.55, 95% CI -4.31 to -0.78, p = 0.005) and the Douleur Neuropathique 4 Questions questionnaire (MD = -1.08, 95% CI -2.05 to -0.11, p = 0.03). By 6 months, pain outcomes were statistically comparable between groups. Hemodynamic variables and complications were comparable between groups.

**Conclusion:**

PSPB and ESP block resulted in similar overall opioid consumption among women undergoing mastectomy. However, PSPB was associated with lower postoperative tramadol consumption.

## Introduction

Breast cancer remains the most prevalent cancer affecting women worldwide.^1^ Modified radical mastectomy is a surgical procedure commonly employed in breast cancer treatment with high success rates.^2^ The procedure removes the entire breast with axillary evacuation and a substantial portion of skin, resulting in a sizeable wound area. Multiple nerves are present in the breast area.^2^ Thus, the procedure can often injure the sensory system surrounding the surgical site.^3^ As a result, a large proportion of breast cancer patients experience moderate or severe acute postoperative pain that cannot be effectively relieved by the diversified analgesic armamentarium available.^4^ The pain greatly hinders early postoperative recovery. In the medium to long-term following surgery, 2% to 78% of the patients suffer from Post-Mastectomy Pain Syndrome (PMPS),^5^ a complex condition characterized by significant and persistent pain lasting for several months.^6^ This condition can restrict normal function, diminishing the patient's overall quality of life and worsening clinical outcomes.^7^

Among the different characteristics potentially associated with the incidence of PMPS, the presence of moderate to severe acute pain in the postoperative period has been a consistent risk factor.^8^ In fact, unresolved acute pain can activate several mechanisms not only at the periphery (primary hyperalgesia) but also within the central nervous system with sensitization of nociceptive neurons (secondary hyperalgesia), inflammation, defective inhibition of nociceptive inputs and higher expression of transmitters, receptors, and ion channels, all of which favor the transition from acute to chronic pain.^9^

In recent years, regional anesthesia techniques have gained prominence for their potential to provide superior pain relief while minimizing opioid consumption and associated adverse effects. Among these techniques, the Pectoserratus Plane Block (PSPB)^10^ and Erector Spinae Plane (ESP)^11^ block have emerged as promising options for thoracic analgesia in breast surgery. Despite increasing use of these regional anesthesia techniques, robust comparative evidence regarding their efficacy and safety for mastectomy remains limited. Previous research has predominantly focused on immediate postoperative outcomes,^12^ limiting the ability to draw comprehensive conclusions regarding long-term pain management and complications. Thus, there is a critical need for additional randomized controlled trials to determine the comparative effectiveness of PSPB and ESP block in this patient population.

To address this gap in existing research, we conducted a single-center, single-blind randomized trial to evaluate the perioperative and long-term outcomes of PSPB versus ESP block in elective mastectomy. Our study aimed to provide comprehensive insights into perioperative opioid consumption, pain intensity, hemodynamic variables, and complication rates, and long-term pain outcomes up to 6 months postoperatively. We hypothesized that PSPB would reduce perioperative opioid consumption and lower chronic pain scores at 3 and 6 months compared to ESP block.

## Material and methods

### Trial registration and design

This was an investigator-initiated, single-center, single-blind, superiority randomized trial with a 1:1 allocation ratio conducted at the Hospital de Base, a tertiary teaching hospital in the Distrito Federal, Brazil. The trial was approved by the local ethics committee (Fundação de Ensino e Pesquisa em Ciências da Saúde, FEPECS, ID: CAAE 38892320.8.0000.0025) and was registered on clinicaltrials.gov (NCT05069805). All patients provided informed consent, and the trial followed the ethical standards of the Declaration of Helsinki.

### Participants

We enrolled female patients aged between 18 and 70 years submitted to elective mastectomy. Patients had to have an American Society of Anesthesiology (ASA) status between I and III. Patients with a previous history of chronic pain, severe cardiac, hepatic, renal diseases, or neurological disorders were excluded. We also excluded pregnant participants, those using psychoactive medications, those taking any medication investigated in the study or patients with known allergies to any of the study drugs.

### Interventions

All procedures and blocks were performed by the study investigators, who were second- and third year anesthesiology residents, under direct supervision of consultant anesthetists with established expertise in thoracic wall regional anesthesia. Investigators received structured, hands-on training in both block techniques prior to study commencement. No blocks were performed by clinicians other than the designated study team.

### Standard procedures for both interventions

Before receiving the interventions (PSPB or ESP block), all patients received standard monitoring, including electrocardiography, pulse oximetry, Non-Invasive Blood Pressure (NIBP), and body temperature. After venous catheterization in the upper limb contralateral to the surgery using an 18G or 20G needle catheter, all patients received Intravenous (IV) midazolam 0.03 mg.kg^-1^ and IV fentanyl 1 mcg.kg^-1^ for anxiolysis, along with 4 mg of IV dexamethasone. All patients underwent general anesthesia, initiated with induction with IV fentanyl 3 mcg.kg^-1^, lidocaine 2 mg.kg^-1^, propofol 2 mg.kg^-1^, cisatracurium 0.15 mg.kg^-1^, or rocuronium 0.6 mg.kg^-1^ at the discretion of the anesthesiologist. Induction was followed by direct laryngoscopy using a Macintosh blade number 3 or 4, and insertion of a 7.0 or 7.5 endotracheal tube with a cuff. Tube position was confirmed by auscultation of lung fields and capnography. Anesthesia was maintained with sevoflurane (approximately 1 to 1.5 Minimum Alveolar Concentration [MAC] of expired fraction) and neuromuscular blockade at the discretion of the anesthesiologist. Increases in Systolic Blood Pressure (SBP) exceeding 20% of the baseline values, measured post-anxiolysis, were interpreted as pain, and increments of 1 mcg.kg^-1^ of fentanyl were administered for each episode, a decision endorsed by two clinical investigators. Reductions in SBP over 20% of baseline or below 90 mmHg were managed with ephedrine 5 to 10 mg. Heart Rate (HR) reductions below 50 bpm associated with a concurrent decrease in SBP were managed with atropine 0.5 to 1 mg. After receiving sedoanalgesia, patients allocated to the ESP block underwent antisepsis with 70% ethanol at the puncture site on the ipsilateral side to be operated on.

### Pectoserratus plane block (PSPB)

PSPB involves two injections: the first between the pectoralis major and minor muscles (priorly known as PECS I), and the second (priorly known as PECS II) between the pectoralis minor and serratus anterior muscles at the level of the third rib. This technique targets the medial and lateral pectoral nerves, the intercostobrachial nerve, the long thoracic nerve, and the thoracodorsal nerve, providing analgesia to the anterolateral chest wall and axilla. Anatomical studies have shown that the conventional PECS II block produces extensive spread to the axillary region, reliably staining the intercostobrachial, thoracodorsal, long thoracic, and both pectoral nerves, which is particularly advantageous for mastectomy with axillary dissection. In contrast, a subserratus approach, where local anesthetic is deposited deep to the serratus anterior, results in limited axillary spread and incomplete blockade of the pectoral nerves, reducing its effectiveness for axillary procedures.

Patients were positioned in the supine position with the arm abducted at 90 degrees. A high-frequency linear ultrasound probe (SonoSite M-Turbo®), protected with a sterile plastic cover, was applied. The probe was positioned in the region below the clavicle, in the deltopectoral groove, identifying the pectoral muscles along with the axillary artery and vein at the level of the first rib. Subsequently, the probe was displaced distally to the space between the 2^nd^ and 3^rd^ ribs, identifying the structures of the pectoralis major, pectoralis minor, and serratus anterior muscles, arranged in sonoanatomy in that order from superficial to deep. A UniPlex NanoLine® 22G × 50 mm needle was introduced in-plane. The entire needle trajectory was visualized during the puncture. A 10 mL injection of 0.5% ropivacaine was administered between the pectoralis major and minor muscles. Needle progression continued to the interfascial plane of the pectoralis minor and serratus anterior muscles with an injection of 20 mL of 0.5% ropivacaine.

### Erector spinae plane (ESP) block

The ESP block is performed by injecting local anesthetic deep into the erector spinae muscle at the level of the transverse process, typically at T4‒T5 for breast surgery. The injectate spreads cranio-caudally along the fascial plane, with potential extension into the paravertebral and epidural spaces, as well as lateral spread into the intercostal spaces. This results in a multidermatomal sensory block involving the dorsal and ventral rami of the thoracic spinal nerves, providing analgesia to the hemithorax, including the breast and, to a lesser extent, the axilla. Imaging and cadaver studies confirm that the ESP block produces extensive craniocaudal spread (spanning 5‒9 thoracic levels) and may reach the paravertebral and neural foraminal spaces; however, the degree of anterior and lateral spread to the axilla and pectoral nerves is less consistent when compared to the PECS II block.

Patients were positioned in a seated position with the support of assistants. A high-frequency linear ultrasound probe (SonoSite M-Turbo®) was applied and protected with a sterile plastic cover. The probe was positioned in the paravertebral region for the identification of the transverse process of the T5 vertebra, the structures of the trapezius, rhomboid, and erector spinal muscles, arranged in sonoanatomy from superficial to deep. A UniPlex NanoLine® 22G × 50 mm needle was introduced in-plane, directed toward the transverse process of the vertebra. The entire needle trajectory was visualized during the puncture. A 30 mL injection of 0.5% ropivacaine was administered between the transverse process and the erector spinal muscles, with the observation of the dispersion of the local anesthetic in this plane.

### Rescue analgesia

In both groups, during the initial 24 hours postoperatively, patients reporting moderate pain (above 2 on a 0-to-10 numeric rating scale) received intravenous paracetamol (acetaminophen) (1 g) as the first analgesic drug every 6 hours. In the case of persistent pain, intravenous tramadol (100 mg) was administered every 6 hours. Patients exhibiting nausea and/or vomiting received intravenous ondansetron (8 mg) every 8 hours. Clinicians responsible for rescue analgesia decisions were blinded to group treatment.

### Block failure

Block failure was defined as the inability of regional anesthesia to provide adequate surgical anesthesia, attributable to either technical errors during administration or patient-specific anatomical variability. Clinically, block failure manifested as nociceptive response at the surgical incision site. The primary hemodynamic correlates of block failure were intraoperative tachycardia and hypertension, operationally defined as a ≥ 20% increase in heart rate and arterial blood pressure relative to pre-induction baseline values.

### Outcomes

The primary outcome was a composite outcome, defined as any use of opioids either during the surgery (fentanyl) or postoperatively (tramadol). Secondary short-term efficacy outcomes included overall pain intensity at rest and incidental pain (pain during movement). Pain intensity was assessed at arrival to and discharge from the post-anesthetic care unit (PACU), and 12 and 24 hours postoperatively. Pain intensity was captured on a 0-to-10 numeric rating scale, with 0 indicating “no pain at all” and 10 representing the “worst possible pain”.

Perioperative hemodynamics and surgery-related outcomes included mean arterial pressure (mmHg), and heart rate (in beats per minute). Additionally, we estimated the expired fraction of sevoflurane intraoperatively, consumption of fentanyl, ephedrine or atropine during surgery, and the use of analgesics (tramadol or dipyrone) and antiemetics (ondansetron) up to 24 hours postoperatively. We assessed the degree of bleeding in the surgical field, which was scored by the surgical team, using a 6-point scale (0 = no bleeding; 1 = slight bleeding with no suction required; 2 = slight bleeding not treating the surgical site but requiring occasional suction; 3 = slight bleeding that improves for several seconds once suction has occurred; 4 = moderate bleeding hampering visualization and requiring frequent suctioning; and 5 = severe bleeding requiring constant suctioning). We graded the level of satisfaction of the surgical team with the surgical procedure and the patient's level of overall satisfaction with perioperative care using a 5-point Likert scale (1 = very dissatisfied, 2 = dissatisfied, 3 = somewhat satisfied, 4 = satisfied, or 5 = very satisfied).

Long-term pain outcomes included the validated, Brazilian Portuguese version of the Douleur Neuropathique en 4 Questions (DN4q), ranging from 0 to 10, with scores ≥ 4 denoting suggestive neuropathic pain.^13^ We also assessed the Short-Form McGill Pain Questionnaire (global, sensory and affective) to capture the quality of pain in the long term using the validated, Brazilian Portuguese version (0 to 45, with higher scores meaning greater pain intensity and unpleasantness).^14^ Chronic pain at the surgical site or adjacent areas was defined as persistent/recurrent pain lasting ≥ 3 months.^15^ Secondary safety outcomes included nausea and vomiting up to 24 hours after surgery.

### Randomization and allocation concealment

Participants were randomly allocated to PSPB or ESP block groups using simple randomization according to a computer-generated random sequence (1:1) (https://www.randomizer.org). The randomization schedule remained concealed from all investigators until the beginning of surgical procedures. The allocation group was communicated to the surgical team just before starting the surgery. Sealed, sequentially numbered opaque envelopes were provided to the operating room. The surgical team opened the envelope at the time of the procedure and implemented the intervention as allocated.

### Blinding

The trial was single blinded at the participant level, with the nerve block performed under sedation. Given the different characteristics of the interventions, blinding of clinical investigators was not possible. To mitigate performance bias, we ensured that all procedures related to general anesthesia and surgery were meticulously followed according to protocol to ensure standardized procedures. We also reduced the risk of detection bias by employing blinded outcome assessors during follow-up assessments.

### Sample size

Based on our prior clinical trial,^15^ and additional local data, we estimated that the primary outcome would be observed in 30% of patients in the PSPB group. We expected the ESP group to be associated with a higher risk of any opioid consumption during and after surgery, with a relative risk of approximately 2.0, resulting in an estimated 60% of participants in the ESP group requiring fentanyl during surgery and/or tramadol postoperatively. Using a two-sample proportions *Z*-test (without continuity correction)^16^ with a 5% alpha level, we calculated that 84 participants (42 per group) would be required to give the trial 80% statistical power (see Supplementary Material for details). To account for attrition (estimated to be approximately 14%), we increased the number to 98 participants (49 per group).

### Statistical analysis

We summarized continuous variables with an approximately normal distribution using means (Standard Deviation, SD). Continuous variables with non-normal distribution were presented as median (Interquartile Range, IQR). Categorical variables were summarized as numbers (percentages). For continuous variables measured at a single time point, we employed Student's *t*-tests for independent groups. In cases where the variables exhibited non-normal distributions, we used a bootstrap *t*-test with 5000 simulations.

Continuous outcomes with repeated measurements were analyzed via linear mixed-effects models using the restricted maximum likelihood estimator for the variance components. Missing data were assumed to be missing at random. In the fixed-effect part of the model, we included treatment, time, and the interaction term between time and treatment. Time was treated as a categorical variable. The random-effects component involved a random intercept, which accounted for the repeated measurements and correlation between time points. Within-group effects were presented as means (95% Confidence Intervals) and treatment effects as Mean Differences (MD) with 95% CI. MDs < 0 favor of the PSPB group.

For binary outcomes evaluated at a single time point, we assessed treatment effects using a generalized linear model with Poisson distribution, log link and robust standard errors. Results were presented as relative risks (95% CI), with RRs < 1 favoring the PSPB group. For binary outcomes with repeated measurements, we used mixed-effects logistic regression using the same predictors as described above for the continuous case. In the mixed-effects models, we evaluated statistical differences at each relevant time point and examined the overall difference between groups (joint test). Categorical outcomes were assessed via Fisher’s exact test for 2xk tables. No corrections for multiple testing were applied because all secondary outcomes were considered exploratory. Statistical analyses were performed using Stata 18 (StataCorp, TX, USA). A p-value < 0.05 (two-tailed) was considered statistically significant.

## Results

### Characteristics of the participants

**Figure 1:**
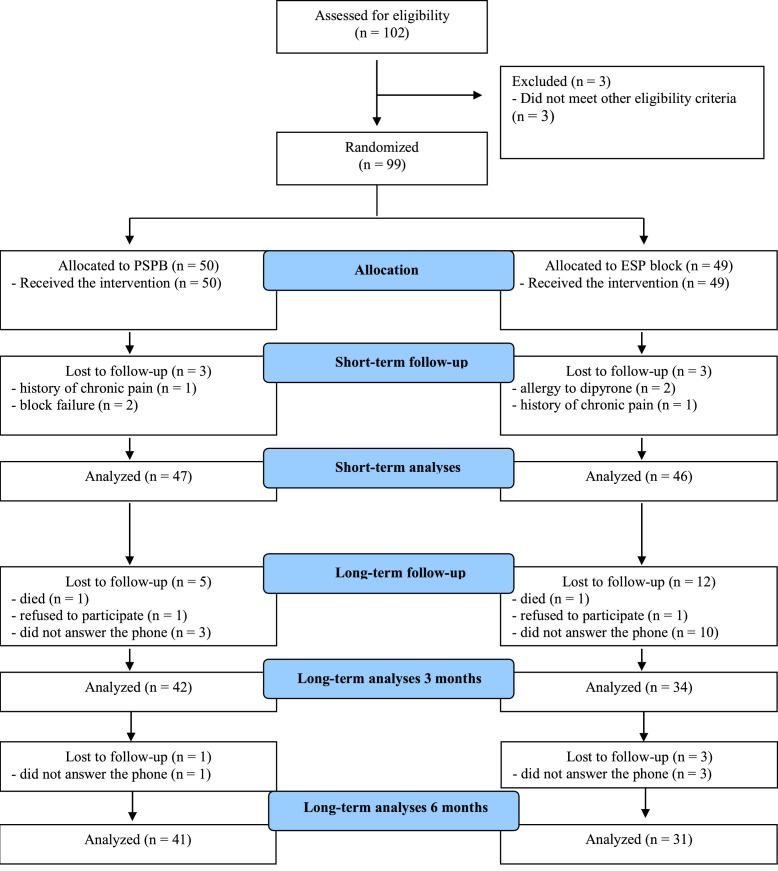
Flowchart summarizing the participant selection process. PSPB, Denotes Pectoserratus Plane Block. ESP, Denotes Erector Spinae Plane Block.

From October 1, 2021, to August 30, 2023, we enrolled 102 patients, of which 99 met all eligibility criteria and were randomized to PSPB (n = 50) or ESP block (n = 49) groups (Fig. 1). Baseline data were evaluated for 97 participants (50 in the PSPB group and 47 in the ESP block group). A total of 93 patients (91%) had complete data regarding the primary outcome (47 in the PSPB group and 46 in the ESP block group). After 3 months of follow-up, 42 patients in the PSPB group and 34 patients in the ESP group had complete data, and after 6 months, 41 patients in the PSPB group and 31 patients in the ESPB group had complete data; patients lost to follow-up were those who did not respond to the telephone call. Throughout the entire study follow-up period, one patient died in each group (see Fig. 1 for details).

The mean (SD) age of the participants was 557 (12) years, and the mean (SD) BMI (kg.m^-2^) was 27.4 (4.3). Sector resection was the most common procedure, performed on 57 (59%) patients. Table 1 presents additional baseline sociodemographic and clinical characteristics, indicating comparable groups at baseline. Both groups had similar anesthesia or surgery durations. However, we observed clinically important differences in the proportion of axillary dissections between the PSPB and ESP block groups (23/47, 48.9% vs. 10/46, 21.7%, respectively).

**Table 1:** Baseline characteristics of the study population.

	PSPB (n = 47)	ESP block (n = 46)
**Sociodemographics**		
Age (years), mean (SD)	55.5 (11.7)	55.8 (12.5)
Weight (kg), mean (SD)	69.6 (12.5)	69.8 (12.0)
BMI (kg.m^-2^), mean (SD)	27.2 (4.3)	27.4 (4.4)
**Clinical characteristics**		
ASA status, n (%)		
I	1 (2)	1 (2)
II	42 (89)	39 (84.7)
III	4 (8.5)	6 (13)
Type-II diabetes, n (%)	9 (19)	11 (23.9)
Obesity, n (%)	16 (34)	13 (28)
Depression or anxiety, n (%)	12 (25.5)	10 (21.7)
Smoking, n (%)	3 (6.5)	5 (10.8)
**Surgery-related characteristics**		
Type of surgery, n (%)		
Mastectomy	3 (6.4)	1 (2)
Radical mastectomy	16 (34)	17 (37)
Sectorectomy	28 (59.5)	28 (61)
Reconstruction, n. (%)	5 (10.6)	2 (43.5)
Axillary dissection, n (%)	23 (48.9)	10 (21.7)^a^
Chemotherapy, n (%)	26 (55.3)	14 (30.4)
Radiotherapy, n (%)	17 (36.1)	22 (47.8)
Surgical time (min), median (IQR)	125 (95‒160)	110 (90‒140)
Anesthesia duration (minutes), median (IQR)	150 (135‒200)	150 (135‒180)

a p-value < 0.05 (two-tailed) was considered statistically significant. BMI, Body Mass Index; ASA status, American Society of Anesthesiologists physical status; ESP, Erector Spinae Plane block; PSPB, Pectoserratus Plane Block; SD, Standard Deviation; IQR, Interquartile Range. Obesity was as a Body Mass Index (BMI) above 30 kg.m^-2^.

### Primary outcome: use of opioids during surgery and postoperatively

Use of either fentanyl intraoperatively or tramadol postoperatively was necessary for 20 of 47 patients (43%) in the PSPB group and 28 of 46 patients (61%) in the ESP block group (RR = 0.70, 95% CI 0.47 to 1.05, p = 0.09).

### Secondary outcomes (intraoperative)

#### Use of fentanyl intraoperatively

Use of fentanyl intraoperatively was necessary for 18 of 47 patients (38%) in the PSPB group and 15 of 46 patients (33%) in the ESP block group (RR = 1.17, 95% CI 0.67 to 2.04, p = 0.57). Among participants who received fentanyl, the mean (SD) dose of fentanyl was statistically significantly lower in the PSPB group than in the ESP block group (mean difference: -28.3 μg, 95% CI -46.6 to -10.1 μg, p = 0.003) (Table 2).

**Table 2:** Short-term clinical and resource utilization outcomes.

**Outcome**	**PSPB** **(n = 47)**	**ESP block** **(n = 46)**	**Mean difference or relative risk (95% CI)**	**p**
**Primary outcome**				
Use of fentanyl intraoperatively and/or tramadol postoperatively, n (%)	20 (43)	28 (61)	0.70 (0.47 to 1.05)	0.09
**Secondary outcomes ‒ intraoperative**				
Use of fentanyl intraoperatively, n (%)	18 (38)	15 (33)	1.17 (0.67 to 2.04)	0.57
Dose of fentanyl (μg), mean (SD)	41.7 (19.2)	70 (31.6)	-28.3 (-46.6 to -10.1)	0.003^a^
Use of ephedrine, n (%)	24 (51)	27 (59%)	0.87 (0.60 to 1.26)	0.46
Dose of ephedrine (mg), mean (SD)	13.5 (7.2)	15.2 (8.3)	-1.7 (-6.04 to 2.66)	0.44
Use of atropine, n (%)	0	0	‒	‒
**Secondary outcomes – postoperatively**				
Use of tramadol	5 (11)	16 (35)	0.31 (0.12 to 0.77)	0.01^a^
Dose of tramadol (mg), mean (SD)	120 (44.8)	125 (57.7)	-5 (-64.2 to 54)	0.86^b^
Time to require tramadol (min), mean (SD)	204 (131.5)	247.8 (154.1)	-43.8 (-204 to 117)	0.57^b^
Use of dipyrone	18 (38)	28 (64)	0.60 (0.39 to 0.92)	0.02^a^
Dose of dipyrone (mg), mean (SD)	1382.4 (740)	1428.6 (825)	-46.2 (-501 to 408)	0.85^b^
Time to require dipyrone (min), mean (SD)	434.1 (278.7)	305 (260)	129.1 (-33.7 to 292)	0.12^b^
Use of ondansetron	7 (15)	10 (22)	0.69 (0.28 to 1.65)	0.40
Dose of ondansetron (mg), mean (SD)	13.7 (6.05)	8.8 (2.53)	4.9 (0.40 to 9.42)	0.03^a^
PONV, n (%)	6 (13)	8 (17)	0.73 (0.27 to 1.96)	0.54
Time in the PACU (minutes), mean (SD)	143.1 (51.5)	111.9 (60.8)	31.1 (6.9 to 55.1)	0.01^a^^,^^b^
Length of hospital stay (hours), mean (SD)	21.8 (3.6)	22.8 (4.0)	-0.94 (-2.52 to 0.63)	0.24^b^
Complication, n (%)^c^	1 (2.1)	1 (2.2)	0.98 (0.06 to 15.4)	0.99

a p-value < 0.05 (two-tailed) was considered statistically significant.

b Bootstrapped *t*-test for independent samples.

c One patient of each group developed an early hematoma, with was managed conservatively without additional interventions. ASA, Status denotes the American Society of Anesthesiologists physical status; ESP, denotes Erector Spinae Plane, PSPB, denotes Pectoserratus Plane Block; PACU, denotes Post-Anesthetic Care Unit; SD, denotes Standard Deviation; SF-MPQ, denotes Short-form McGill Pain Questionnaire.

#### Intraoperative mean arterial pressure and heart rate

**Figure 2:**
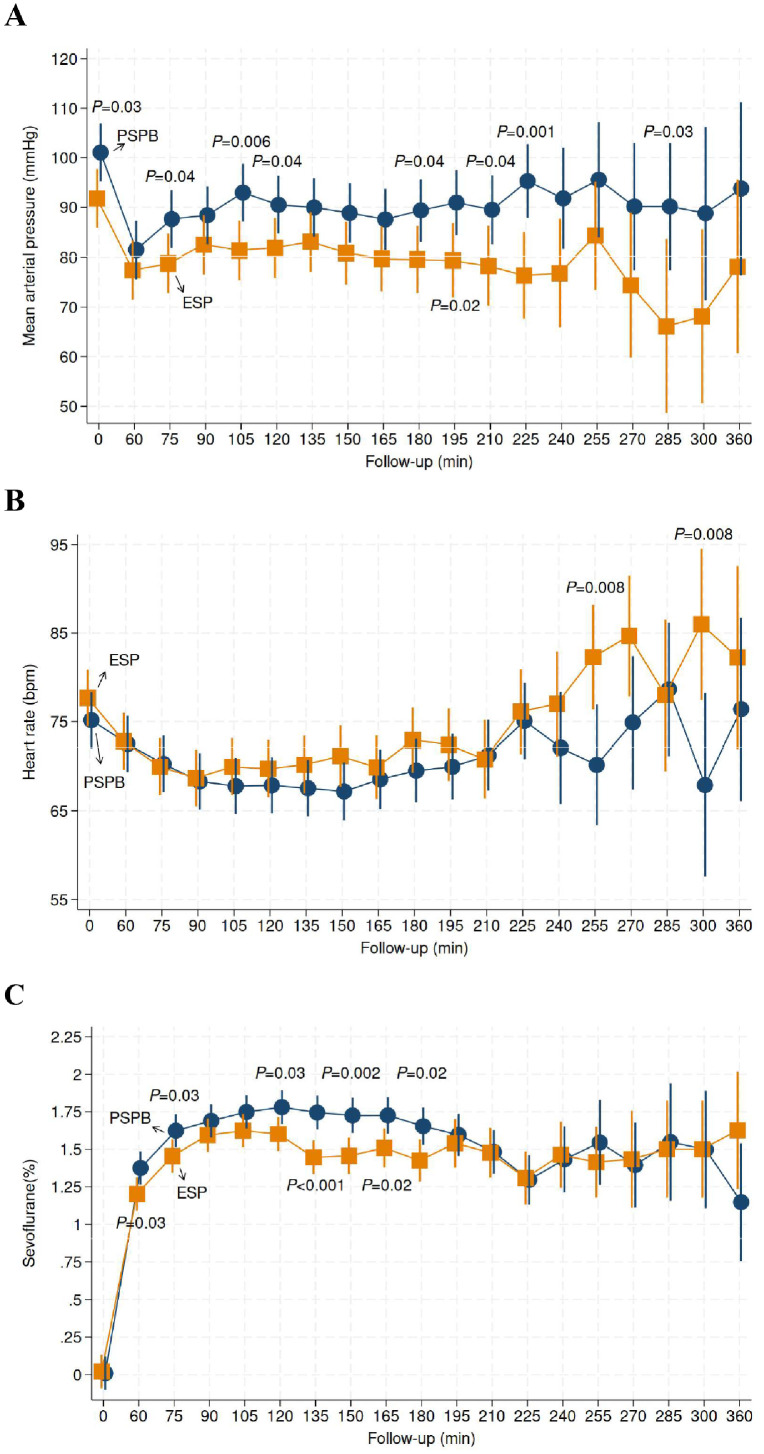
Intraoperative trajectories of mean arterial blood pressure (mmHg) (panel A), heart rate (bpm) (panel B) and sevoflurane consumption (%) (panel C). PSPB denotes pectoserratus plane block (blue disks). ESP denotes erector spinae plane block (orange squares). Results are presented as means (95% Confidence Intervals) and were obtained via mixed-effects linear regression models.

Figure 2 (panel A) shows the variations in mean arterial pressure between the PSPB and ESP block groups throughout the surgical procedures. Although statistical differences occasionally emerged between the two groups at certain specific time intervals, no consistent pattern was observed. The joint test indicated that the two groups were not statistically different overall (p = 0.27). Similar results were observed for heart rate (p = 0.20) (Fig. 2, panel B).

#### Sevoflurane consumption

Figure 2 (panel C) shows a significant difference in sevoflurane consumption between groups (joint test, p = 0.003), particularly between minutes 75 and 180 during surgery, with the PSPB group consuming more sevoflurane than the ESP block group.

#### Use of ephedrine and atropine

Use of ephedrine intraoperatively was necessary for 24 of 47 patients (51%) in the PSPB group and 27 of 46 patients (59%) in the ESP block group (RR = 0.87, 95% CI 0.60 to 1.26, p = 0.46). Among participants who received ephedrine, the mean (SD) dose of ephedrine was not statistically different between the groups (mean difference: -1.7 mg, 95% CI -6.04 to 2.66 mg, p = 0.44) (Table 2). None of the patients required atropine in our study.

#### Blood volume loss

No statistical differences were observed between the groups regarding blood volume loss (Table S1, p = 0.65).

#### Surgeon satisfaction

The surgical approach was associated with surgeon satisfaction (p < 0.001), with surgeons in the ESP block group choosing higher satisfaction categories than those from the PSPB group (Table S1).

### Secondary outcomes – postoperatively (short-term)

#### Use of tramadol postoperatively

Use of tramadol for pain control after surgery was necessary for 5 of 47 patients (11%) in the PSPB group and 16 of 46 patients (35%) in the ESP block group (RR = 0.31, 95% CI 0.12 to 0.77, p = 0.01). Among patients requiring tramadol postoperatively, there was no difference in the mean (SD) dose between the PSPB and ESP block groups (120 [44.8] vs. 125 [57.7] mg, respectively; p = 0.86), with a median (IQR) dose of 100 (100 to 100) mg for both groups.

#### Use of dipyrone postoperatively

Use of dipyrone postoperatively was necessary for 18 of 47 patients (38%) in the PSPB group and 28 of 44 patients (64%) in the ESP block group (RR = 0.60, 95% CI 0.39 to 0.92, p = 0.02). Among patients receiving dipyrone postoperatively, there was no difference in the mean (SD) dose between the PSPB and ESP block groups (1382 [740] mg vs. 1429 [825] mg, respectively; p = 0.85), with a median (IQR) dose of 1000 (1000 to 2000) mg for both groups.

#### Time to request analgesics postoperatively

There was no statistically significant difference in time to request for analgesics between the groups, regardless of whether tramadol or dipyrone was used (Table 2).

#### Use of antiemetics

Use of ondansetron postoperatively was necessary for 7 of 47 patients (15%) in the PSPB group and 10 of 46 patients (22%) in the ESP block group (RR = 0.69, 95% CI 0.28 to 1.65, p = 0.40). Among participants who received ondansetron, the mean (SD) dose of ondansetron was statistically significantly higher in the PSPB group than in the ESP block group (mean difference: +4.9 mg, 95% CI 0.40 to 9.42 mg, p = 0.03), but the clinical relevance of this difference is unclear (Table 2). There was no statistical difference in the risk of developing postoperative nausea and vomiting between the groups (RR = 0.73, 95% CI 0.27 to 1.96, p = 0.54).

#### Pain intensity ‒ short-term

**Figure 3:**
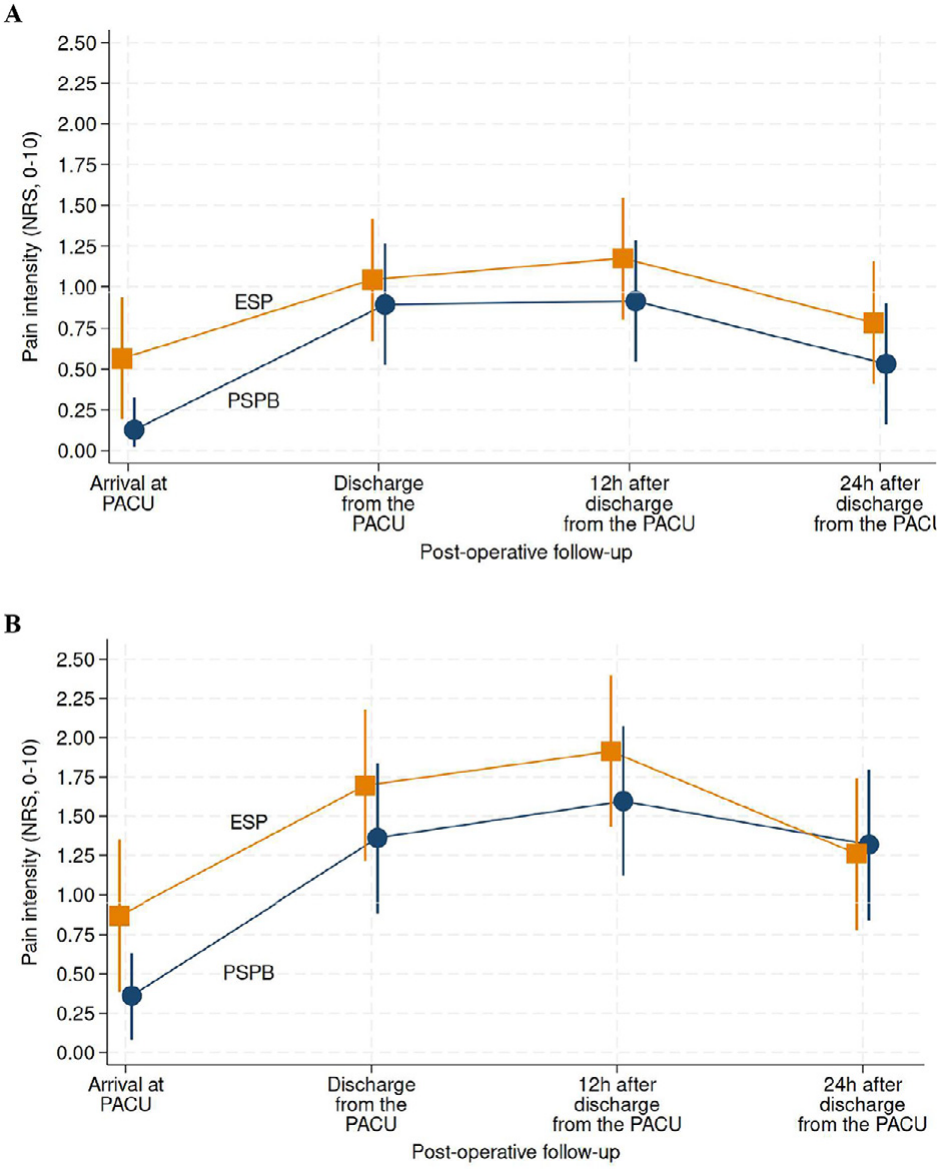
Trajectories of short-term pain intensity. In panel A, pain intensity at rest (panel A). In panel B, incidental pain (pain intensity under stress/movement). NRS denotes numeric rating scale. PSPB denotes Pectoserratus Plane Block (blue disks). ESP denotes Erector Spinae Plane block (orange squares). There was not statistically significant between-group difference observed at any time point, either in terms of pain at rest or pain under stress (movement). The joint test resulted in a p-value of 0.55 for pain at rest, and a p-value of 0.50 for incidental pain.

Figure 3 (panels A and B) shows the trajectory of pain intensities from PACU arrival to 24 hours post-discharge. Pain at rest (panel A) or incidental pain (panel B) did not differ statistically between the two groups.

#### Time in the PACU and length of hospital stay

The time in the PACU was statistically significantly higher for the PSPB group than for the ESP block group (mean difference: 31.1 minutes, 95% CI 6.9 to 55.0, p = 0.01). However, there was no statistical difference between the average length of hospital stay for the groups (mean difference: -0.94 hours, 95% CI -2.52 to 0.63, p = 0.24) (Table 2).

#### Patient satisfaction and complications

Patients in the PSPB group were associated with higher levels of satisfaction compared to their ESP-treated counterparts (p = 0.04) (Table S2). However, the overall satisfaction rates were high in both groups, indicating that these differences are unlikely to have clinical significance. In each group, one patient experienced a complication, with no statistically significant difference between the groups (Table 2).

### Secondary outcomes ‒ long-term

#### Pain intensity and global chronic pain ‒ long-term

**Figure 4:**
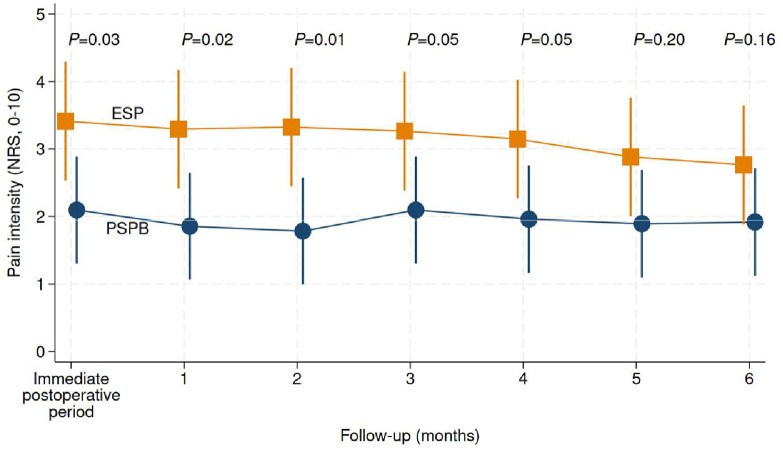
Trajectories of long-term pain intensity. PSPB denotes Pectoserratus Plane Block (blue disks). ESP denotes Erector Spinae Plane block (orange squares). The immediate postoperative period refers to the assessment of pain intensity within a few days following surgery, typically ranging from 1 to 2 days after the procedure. The joint test resulted in a p-value of 0.24 (linear mixed-effects model).

Figure 4 shows the trajectory of pain intensity from the immediate postoperative period to 6 months after surgery. Pain intensity was statistically significantly lower for the PSPB group than for the ESP block group from the immediate postoperative period to two months after surgery. However, when considering all time points together, there were no significant statistical differences between the groups (joint test, p = 0.24). Based on responses regarding the duration of pain, 22 of 42 patients (52%) in the PSPB group and 27 of 34 patients (79%) in the ESP block group were classified as experiencing chronic pain at 3 months (RR = 0.66, 95% CI 0.47 to 0.92, p = 0.02). However, at 6 months, the groups were statistically similar regarding the proportion of patients classified as experiencing chronic pain (20 of 41 [49%] vs. 18 of 31 [58%] for the PSPB and ESP block groups, respectively, RR = 0.84, 95% CI 0.54 to 1.30, p = 0.43) (Table 3).

**Table 3:** Secondary medium- and long-term outcomes (pain-related).

Outcome	PSPB	ESP block	Mean difference or relative risk (95% CI)	p
Secondary outcomes ‒ pain-related				
Chronic pain, n (%)				
3 months^b^	22 (52)	27 (79)	0.66 (0.47 to 0.92)	0.02^a^
6 months^c^	20 (49)	18 (58)	0.84 (0.54 to 1.30)	0.43
Use of rescue medications, n (%)				
3 months^b^	13 (31)	17 (51.5)	0.61 (0.35 to 1.07)	0.09
6 months^c^	10 (24)	13 (41)	0.59 (0.31 to 1.15)	0.12
DN4q scores, mean (SD)				
3 months^b^	2.30 (2.37)	3.38 (2.40)	-1.08 (-2.05 to -0.11)	0.03^a^
6 months^c^	1.80 (1.87)	2.30 (2.10)	-0.47 (-1.46 to 0.52)	0.35
Neuropathic pain, n (%)				
3 months^b^	15 (36)	18 (53)	0.68 (0.41 to 1.15)	0.15
6 months^c^	11 (27)	12 (39)	0.66 (0.34 to 1.27)	0.21
SF-MPQ, mean (SD)				
Global				
3 months^b^	3.2 (3.6)	5.8 (5.3)	-2.55 (-4.32 to 0.78)	0.005^a^
6 months^c^	2.4 (3.0)	3.4 (3.7)	-1.27 (-3.07 to 0.53)	0.17
Sensory				
3 months^b^	2.8 (3.18)	4.97 (4.34)	-2.16 (-3.70 to -0.62)	0.006^a^
6 months^c^	2.34 (2.84)	3.35 (3.52)	-1.09 (-2.66 to 0.48)	0.18
Affective				
3 months^b^	0.40 (0.86)	0.79 (1.91)	-0.39 (-0.94 to 0.16)	0.17
6 months^c^	0.1 (0.37)	0.06 (0.36)	-0.21 (-0.77 to 0.35)	0.46

a p-value < 0.05 (two-tailed) was considered statistically significant.

b Based on 42 participants with complete data in the PSPB group and 34 in the ESP.

c Based on 41 participants with complete data in the PSPB group and 31 in the ESP. DN4q, Douleur Neuropathique 4 Questions; ESP, denotes Erector Spinae Plane block; PSPB, Pectoserratus Plane Block; SD, Standard Deviation; SF-MPQ, Short-form McGill Pain Questionnaire.

#### Rescue medications and chronic pain-related questionnaires

The PSPB and ESP block groups showed no difference in the proportions of patients requiring rescue medication for pain at either 3 or 6 months after surgery (Table 2). Similar conclusions were drawn regarding DN4q scores and the proportion of patients classified as having neuropathic pain. At 3 months postoperatively, SF-MPQ (global) ratings were significantly lower in the PSPB group compared to the ESP block group (mean difference: -2.55, 95% CI -4.31 to -0.78, p = 0.005). However, at 6 months postoperatively, no significant difference was observed between the groups for this outcome (mean difference: -1.27, 95% CI -3.07 to 0.53, p = 0.17). Results for the subcomponents (sensory and affective) followed similar trends (Table 2).

## Discussion

### Principal findings

This randomized controlled trial showed that PSPB and ESP blocks were comparable in terms of intraoperative and postoperative opioid consumption in patients undergoing elective mastectomy. Both anesthetic blocks were associated with a similar and low risk of complications. However, statistically and clinically important differences emerged 3 months after surgery, with PSPB-treated patients exhibiting a reduced risk of chronic pain, lower DN4q scores, and lower pain intensity scores than their ESP block-treated counterparts. Nonetheless, at 6 months after surgery there were no noticeable differences in pain intensity or pain quality between women treated with PSPB and those treated with ESP block.

In principle, both techniques are capable of providing good anesthetic conditions for breast surgery, and therefore, similar general anesthetic consumption would be expected. However, individual patient variations, anesthesiologist preference for sevoflurane dosing, and differences in surgical procedures (such as more axillary dissections in the PSPB group, as observed in this study) may have contributed to the statistically significant reduction in sevoflurane consumption in the ESP group. Nevertheless, this reduction was not deemed clinically significant. Notably, most studies have primarily evaluated outcomes related to postoperative analgesia, opioid consumption, and quality of recovery, rather than intraoperative consumption of volatile anesthetics like sevoflurane.

Despite higher reported pain scores and increased postoperative opioid requirements in the ESP block cohort, we observed that ESP block patients were associated with a reduced duration of stay in the PACU. However, the observed difference of approximately 30 minutes in PACU length of stay was not deemed clinically significant, as discharge timing is influenced by multiple variables, including staffing schedules, PACU census, and the circadian timing of patient admission (morning, afternoon, or evening), among other operational factors.

We observed that patient satisfaction was higher in the PSPB group, while surgeon satisfaction was greater with ESP block. Patients reported greater satisfaction with PSPB, likely due to superior analgesic efficacy and reduced opioid consumption. Conversely, from the surgeon’s perspective, local anesthetic spread into the axilla during PSPB can complicate axillary dissection, hinder the use of electrocautery, and particularly impede identification of the sentinel lymph node, which could explain, at least partially, the surgical team’s concerns.

### Comparison with previous studies

We are not aware of previous or ongoing randomized trials of PSPB versus ESP block specifically designed to evaluate the composite use of intraoperative fentanyl and/or tramadol postoperatively as the primary outcome, or their efficacy regarding long-term pain intensity after elective mastectomy. Wong et al. (2021) conducted a comprehensive network meta-analysis and used direct and indirect evidence from 66 randomized trials in breast surgery.^17^ The authors found no evidence of a difference in postoperative pain intensity between the PSPB and the ESP block. However, this analysis focused on immediate postoperative outcomes, such as pain intensity at rest within 0‒2 hours and 8‒12 hours after surgery. Our findings for the short-term pain score were consistent with the results reported by Wong et al. (2021).^17^

A few head-to-head trials corroborate our findings, but they used different local anesthetics for local anesthetic infiltration. For example, Altıparmak et al. (2019) assessed total postoperative tramadol consumption in the first 24 hours after radical mastectomy surgery as the primary outcome.^12^ The authors used 0.25% levobupivacaine for the blocks. This trial demonstrated a statistically significant reduction in tramadol consumption and a lower need for rescue analgesia in the first 24 postoperative hours in the PSPB group compared to the ESP block.^12^ In terms of fentanyl consumption, there were no discernible differences in intraoperative fentanyl usage between the groups.^12^

In another 2019 randomized trial, Sinha et al. used 0.2% ropivacaine in both groups and also demonstrated the analgesic superiority of PSPB over ESP block.^18^ The authors reported a decrease in morphine consumption during the first 24 hours after surgery in the PSPB group compared to the ESP block group, and a 23% longer analgesia duration with the PSPB compared to the ESP block.

In our trial, patients who received PSPB exhibited lower mean scores on the SF-MPQ global scale at 3 months, with a greater reduction in sensory domain scores than for the ESP block, indicating that regional anesthesia techniques may play a more crucial role in modulating nociceptive pain. Previous evidence suggests that the superiority of PSPB, when compared to the ESP block, in managing nociceptive pain could be attributed, at least partially, to its more complete blockade of sensory innervation in the breast.^19^, ^20^, ^21^, ^22^, ^23^ This blockade includes branches from the brachial plexus, lateral cutaneous branches of the intercostal nerve at the mid-axillary line, the long thoracic nerve, and the thoracodorsal nerve, covering the C5‒T4 dermatomes.^19^, ^20^, ^21^, ^22^, ^23^ In contrast, ESP block primarily targets intercostal nerves and, when performed at the level of the fifth vertebral transverse process, extends sensory blockade to the T2‒T9 dermatomes.^19^, ^20^, ^21^, ^22^, ^23^ Notably, PSPB has been associated with decreased levels of stress hormones like cortisol and prolactin postoperatively compared to the ESP block.^24^

### Strengths and limitations

Our study has several strengths, including a comprehensive assessment of resource utilization and clinical outcomes spanning perioperative, short-term, and long-term periods. However, several limitations are worth discussing. First, we did not adjust our results for multiple tests. We considered all secondary outcomes exploratory, which needs further assessment in confirmatory trials.^25^ Second, our sample size was relatively modest, and we cannot rule out small to moderate differences in treatment effects between PSPB and ESP block concerning intraoperative and postoperative opioid consumption. Third, while our results have high internal validity for elective mastectomies, they may not generalize to other non-mastectomy-related surgical procedures. Fourth, our attrition rates were higher than anticipated, particularly 6 months after surgery, which may have reduced our statistical power to detect differences between the groups. Patients were often lost to follow-up due to lack of contact and non-responsiveness. Future trials examining long-term pain after mastectomy should anticipate high attrition rates and incorporate retention strategies to mitigate these challenges. Fifth, participants in the PSPB group more frequently underwent axillary lymph node dissection and immediate breast reconstruction, procedures that could increase intraoperative requirements for sevoflurane, perioperative opioid consumption, and the risk of both acute and persistent postoperative pain. Nonetheless, these anticipated effects were only partially observed. Specifically, sevoflurane consumption was higher intraoperatively in the PSPB group than in the ESP block group, while opioid consumption was lower in the PSPB group. These results suggest that the increased surgical burden did not compromise, but rather reinforced, our confidence in the postoperative analgesic efficacy of PSPB. Sixth, although chronic pain rates estimated in our study were relatively high in both groups when considering the broader definition of chronic pain, the incidence of neuropathic pain, which is of greater clinical concern due to its substantial impact on patients, fell within the range reported in the existing literature on chronic pain following breast cancer surgery.^5^^,^^26^

## Conclusion

We can conclude that both ESP and PSPB blocks are safe, effective, and comparable for oncologic mastectomy. However, PSPB presents a slight advantage in outcomes related to acute and chronic pain, including reduced postoperative opioid consumption and lower incidence of chronic pain. The choice between the two techniques may be based on the type of breast surgery, surgical team experience, and anesthesiology team's technique preference.

## Data availability statement

The datasets generated and/or analyzed during the current study are available from the corresponding author upon reasonable request.

## Presentation

Preliminary data for this study were presented at the Brazilian Anesthesiology Congress in November 2022.

## Conflicts of interest

The authors declare no conflicts of interest.

## Appendix Supplementary materials

Image, application 1

## Footnotes

Supplementary material associated with this article can be found in the online version at doi:10.1016/j.bjane.2025.844691.
