# Supplementary material for: Efficacy of pectoserratus plane block versus erector spinae plane block on acute and chronic pain after mastectomy: randomized clinical trial
Source: Braz J Anesthesiol. 2025 Oct 24;75(6):844691. doi: 10.1016/j.bjane.2025.844691 (PMC12664958; doi:10.1016/j.bjane.2025.844691)
Supplement: Supplementary file 1 [file mmc1.doc]

**BJAN-D-24-00686_Supplementary material**

**Table S1** Surgery-related outcomes.

| **Outcome** | **PSPB**  **(n = 47)** | **ESP block**  **(n = 46)** | **p** |
| --- | --- | --- | --- |
| Bleeding volume, n (%) |  |  |  |
| No bleeding | 4 (8.5) | 5 (10.9) | 0.65 |
| Light bleeding without suction | 41 (87.2) | 37 (80.4) |
| Bleeding with suction | 2 (4.3) | 4 (8.7) |
| Moderate bleeding | 0 | 0 |
| Heavy bleeding | 0 | 0 |
| Satisfaction of the surgical team, n (%) |  |  |  |
| Very dissatisfied | 0 | 0 | < 0.001 |
| Dissatisfied | 3 (6.4) | 0 |
| Neutral | 11 (23.4) | 1 (2.2) |
| Satisfied | 28 (59.6) | 21 (45.6) |
| Very satisfied | 5 (10.6) | 24 (52.2) |

ESP, Denotes Erector Spinae Plane block; PSPB, Denotes Pectoserratus Plane Block.

**Table S2** Patient satisfaction with the surgical procedure.

| **Satisfaction category, n (%)** | **PSPB**  **(n = 47)** | **ESP block**  **(n = 45)** | **p** |
| --- | --- | --- | --- |
| Very dissatisfied | 0 | 0 | 0.04 |
| Dissatisfied | 0 | 0 |
| Neutral | 0 | 2 (2) |
| Satisfied | 8 (17) | 13 (29) |
| Very satisfied | 39 (83) | 28 (62) |

PSPB, Denotes Pectoserratus Plane Block; ESP, Denotes Erector Spinae Plane Block.

**Figure S1** Intraoperative trajectories of mean arterial blood pressure (mmHg) (panel A), heart rate (bpm) (panel B) and sevoflurane consumption (%) (panel C). PSPB denotes Pectoserratus Plane Block (blue disks). ESP denotes Erector Spinae Plane block (orange squares). Results are presented as means (95% Confidence Intervals) and were obtained via mixed-effects linear regression models.

**Supplementary material**

***Sample size determination***

We used the following formula to determine the sample size (*N*) per group: *N* = [ Z_(1-α/2)_ * sqrt(2 * p_m_ * (1 - p_m_)) + Z_(1-β)_ * sqrt(p_c_ * (1 - p_c_) + p_t_ * (1 - p_t_)) ]² / (p_c_ – p_t_)²; where p_t_ denotes the risk in the treatment group, p_c_ the risk in the control group, and p_m_ = (p_t_ + p_c_)/2. Z_(1-α/2)_ is the critical value of the standard normal distribution for a two-sided test at significance level α and Z_(1-β)_ is critical value of the standard normal distribution corresponding to the desired power. For details, see Wittes (2002).[1] We set α at 5% and power at 80%.

**Reference**

1. Wittes J. Sample size calculations for randomized controlled trials. Epidemiologic reviews. 2002;24:39-53.
